# Vocalizations of adult male Asian koels (*Eudynamys scolopacea*) in the breeding season

**DOI:** 10.1371/journal.pone.0186604

**Published:** 2017-10-20

**Authors:** Abdul Aziz Khan, Irfan Zia Qureshi

**Affiliations:** Department of Animal Sciences, Faculty of Biological Sciences, Quaid-i-Azam University, Islamabad, Pakistan; Hungarian Academy of Sciences, HUNGARY

## Abstract

Defining the vocal repertoire provides a basis for understanding the role of acoustic signals in sexual and social interactions of an animal. The Asian koel (*Eudynamys scolopacea*) is a migratory bird which spends its summer breeding season in the plains of Pakistan. The bird is typically wary and secretive but produces loud and distinct calls, making it easily detected when unseen. Like the other birds in the wild, presumably Asian koels use their calls for social cohesion and coordination of different behaviors. To date, the description of vocal repertoire of the male Asian koel has been lacking. Presently we analyzed and described for the first time the vocalizations of the adult male Asian koel, recorded in two consecutive breeding seasons. Using 10 call parameters, we categorized the vocalization type into six different categories on the basis of spectrogram and statistical analyses, namely the; “*type 1 cooee call*”, “*type 2 cooee call*”, “*type 1 coegh call*”, “*type 2 coegh call*”, “*wurroo call*” and “*coe call*”. These names were assigned not on the basis of functional analysis and were therefore onomatopoeic. Stepwise cross validated discriminant function analysis classified the vocalization correctly (100%) into the predicted vocal categories that we initially classified on the basis of spectrographic examination. Our findings enrich the biological knowledge about vocalizations of the adult male Asian koel and provide a foundation for future acoustic monitoring of the species, as well as for comparative studies with vocalizations of other bird species of the cuckoo family. Further studies on the vocalizations of the Asian koel are required to unravel their functions in sexual selection and individual recognition.

## Introduction

Bird calls are functionally rich signals and are used in a wide range of behavioral contexts by passerine and non-passerine birds. In the past recent years, the birds have been rigorously identified with automatic recognition using the audio signals [1], and this approach has become more advanced with time due to the reason that autonomous recording units (ARUs) are affordable, power sufficient and the data can be obtained from distant areas which can be transmitted directly to the laboratory for analysis through GSM or satellite connection [2].

Spectrogram analyses have demonstrated that birds produce two kinds of vocalizations, a) typically short and simple calls, and b) usually lengthy and well-structured organized songs mainly associated with reproduction and aggression [3]. Singing lengthy songs is not the usual feature of non-passerine birds, instead they typically use short and simple calls in a variety of behavioral contexts like interactions between territories [4, 5], coordination of reproductive activities [6], foraging [7], avoidance of predator [8] and contact maintenance [9]. These vocalizations are less complex and characterized by mono syllabic and changing frequency patterns [10]. Ornithological studies are largely dependent on recordings of vocalizations about the subject bird [11]. Compared to passerine birds whose communication behavior is well represented in literature [12, 13], call–based communication system of non-passerine birds has been given less attention in the field of bioacoustics [14, 15].

Birds of the cuckoo family (Cuculidae) are although famous for their loud vocalizations, but unlike Psittaciformes, Passeriformes, and Trochilidae, cuckoos are non-vocal-learners [16, 17]. Learners acquire their vocalizations by imitation [18] and non-learners through inheritance [19], the main functions of vocalizations seem to be similar in both groups, and are involved in mate attraction and deterrence of rival males [20, 21]. Despite the extensive research on cuckoos, the description of the Asian koel’s vocalizations is still lacking. Koels are typically shy and secretive birds but produce loud and distinguishable calls that are easily detectable [22]. Cuckoos produce hundreds of sufficiently loud calls on a daily basis but depending on the habitat, and if the weather is calm, the common cuckoo (*Cuculus canorus*) calls for example, can be heard at a distance of approximately 2–3 km [17].

The Asian koel is a migratory bird that spends its summer in the plains of Pakistan and migrates back towards India as winter begins [23]. It is primarily a brood parasite of mynas and crows all over the Indian subcontinent [24]. In the Pothwar region of Pakistan, its breeding season begins from late May and continues till early September [25]. Its habitat differs from the other family members because, unlike other cuckoo species which are mostly insectivorous, the Asian koel is totally carpophagous (feeds on fruit) and occasionally adds insects to its diet [23, 26]. Asian koels can also be differentiated from other members of the family Cuculidae in that they often tolerate the young of their hosts, rather than forcing them out of the nest [26, 27]. This species lacks sexual dimorphism in body size, but shows prominent sexual dimorphism in plumage color [28].

Maller and Jones [29] previously described three main calls for the adult male koel in their breeding season in Australia and labeled them as “*cooee call*”, “*wurroo call*” and “*whik call*”. Data regarding vocalizations of the Asian koel are scant, and thus the present study was designed to provide a detailed description of vocalizations of the adult male Asian koel during its breeding season. We first categorized the vocal signals on the basis of audio and visual examination of the spectrogram. Subsequently, various spectral and temporal parameters were measured that were used for the statistical validation of the vocal categories. We also noticed some of usages for all the vocal categories in the field. Finally, besides standardized categories as mentioned above, onomatopoeic names were introduced to describe the vocal categories.

## Methods

### Ethics statement

All the recordings were carried out non-invasively to avoid causing disturbance in the daily activities of target birds or any other species. No specific permits were required for the recordings of wild birds in public parks and gardens in Pakistan. The present study was approved by the “Institutional Bio Ethical Committee at Quaid-i-Azam University, (BEC-FBS-QAU-02)”.

### Study site and recordings

Vocalizations were collected in the wild from a total of 62 unmarked adult male Asian koels. The field work was conducted between 2015 and 2016 from April to September in different areas of Islamabad, Capital Territory, Pakistan. Birds were spotted in different parks and gardens throughout the Islamabad area, including the Margalla hills.

Recordings were made using a ME67/K6 directional microphone (Sennheiser, Germany) connected to TASCAM dr-I00 recorder (TEAC Corporation, Japan). The head of microphone was covered with MZW 66 foam windshield (Sennheiser, Germany) to effectively protect against wind and pop-up noise during recording. Instrument settings were kept similar for all recordings. The recordings were made at the sampling rate of 44.1 kHz and 16 bit resolution because this sampling rate and resolution have been demonstrated previously to be sufficient for the extraction of jitter and shimmer [30] and other acoustic parameters.

Recordings of perched koels were made by the authors at dawn (4:30–6:30 a.m., local time) and dusk (17:00–18:30 p.m., local time), as these birds are more active at these times and also because of least signal to noise ratio. Moreover, it was found that at these times, other highly vocal species in this area, the blue-throated barbet (*Megalaima asiatica*) and the Indian tree pie (*Dendrocitta vagabunda*) were usually less active. Calls that were recorded in the noon times often overlapped with vocalizations from other species. Such calls were therefore excluded from the analyses. The bird calls were recorded from their initial arrival dates to Pakistan (28 April 2015 and 5 March 2016) till their migration back to India (16 September 2015 and 24 September 2016). All the recordings were taken during good weather condition (without wind, rain). The vocalizations were recorded at an approximate distance of 10 to 15 meters taking care not to disturb the target bird. Recordings in which the target bird was disturbed either due to the presence of nearby birds or detected the presence of authors and changed its original location approximately more than one meter, such recordings were also ignored. To avoid the recordings of the same bird more than once, we kept approximately half kilometer distance between two recording sites. If the opportunity to record a bird in a site was missed, we ignored that site for recording. The authors also did their best effort to make video recordings, but as these birds were shy and remained hidden in evergreen dense plants, consequently we were unable to make any reliable video recordings.

### Spectrographic analysis

A total of 64 audio recordings were digitized on Dell Inspiron n4030 Core i3 Laptop (Dell Technologies Inc. USA). All the audio files were initially analyzed on the basis of spectrograms for different vocal categories with the help of Cool edit pro Version 2 (Syntrillium Software Corporation, Phoenix, AZ, USA). The contrast and brightness were adjusted to provide clear pictures of the vocalizations and all of the calls were viewed at the 0.5 s zooming level. A total of 750 calls were identified during the initial examination of spectrograms. Further, due to overlapping calls from other birds, or insufficient signal to noise ratio of the target birds, 334 good quality calls were selected from this original dataset of calls. These were categorized as: *type 1 cooee* = 108 (contributed by a total of 18 individuals; 6.0 ± 0.65 per individual), *type 2 cooee call* = 36 (contributed by a total of 6 individuals; 6.0 ± 2.40 per individual), *type 1 coegh call* = 40 (contributed by a total of 7 individuals; 5.71 ± 0.99 per individual), *type 2 coegh call* = 52 (contributed by a total of 9 individuals; 6.5 ± 1.86 per individual), *wurroo call* = 55 (contributed by a total of 8 individuals; 6.87 ± 1.36 per individual), *coe call* = 43 (contributed by a total of 7 individuals; 6.14 ± 0.96 per individual, “mean ± S.E.”) for comprehensive acoustic measurements.

### Acoustic analysis

In the acoustic analysis, we measured 10 call parameters and three categorical call variables for the selected vocalizations which have been used previously in the vocal repertoire studies [31–33]. Measurable variables defined various spectral, temporal, frequency and amplitude parameters (Table 1).

**Table 1:** Abbreviations list and description of various acoustic parameters and categorical call variables measured for each vocal category.

Abbreviations	Description of Parameter
Dur (s)	Total duration of call
F0 s (Hz)	Fundamental frequency value at the start of the call
F0 e (Hz)	Fundamental frequency value at the end of the call
F0 min (Hz)	Minimum fundamental frequency value across the call
F0 max (Hz)	Maximum fundamental frequency value across the call
Pitch m (Hz)	Mean pitch value across the call
Pitch min (Hz)	Minimum pitch value across the call
Pitch max (Hz)	Maximum pitch value across the call
Jitter (%)	The average absolute difference between the consecutive F0 periods frequency values divided by mean F0 frequency value [31].
Shimmer (%)	The average absolute difference between the consecutive F0 periods amplitude values divided by mean F0 amplitude value [31].
No. of full-length SEs	Number of full-length secondary elements across the call
No. of half-length SEs	Number of half-length secondary elements across the call
No. of diffused SEs	Number of diffused secondary elements across the call

All the call parameters and categorical variables mentioned in Table 1 were measured for all calls by using Praat software v. 6.0.20 [34]. Initially all of the calls were selected individually and F0 contour was extracted using the cross correlation method for the spectrograms of calls through Fast Fourier Transform method (FFT), Gaussian window shape, window length of 0.012 s, frequency view range of 700–24000 Hz, pitch floor of 75–700 Hz, dynamic range of 70 dB, intensity range of 30–100 dB. The F0 s (Hz), F0 e (Hz), F0 min (Hz) and F0 max (Hz) values were then measured from the extracted F0 contour. All measurements were made using the semiautomatic method using an onscreen cursor selection by the same person. The F0 contour was extracted according to the method previously described for the vocal repertoire of the African penguins (*Spheniscus demersus*) [31]. To measure F0 contour, each syllable (containing primary and secondary elements) was selected as a whole. To measure the jitter (%) and shimmer (%), [Sound: To Pitch (cc)] command was used. The jitter is the cycle to cycle fluctuation in the fundamental frequency while, the shimmer is the cycle to cycle fluctuation in amplitude [35, 36]. Both are used for speaker verification [37], to study the pathological characteristics of voice [38] and laryngeal pathologies [39, 40]. We also assigned score 1 to a full-length and 0.5 score to a half-length elements in all type of calls. However, in the case of *type 1 coegh calls* and *type 2 cooee calls* it was difficult to score the diffused secondary elements for statistical analysis, hence the scores were assigned on the basis of the number of secondary elements of the nearby *type 1 cooee calls* in the same bout.

### Statistical analysis

All the statistical analyses were performed using IBM SPSS Statistics 20.0 (USA). The data were checked for normality (Kolmogorov-Smirnov test). We transformed the variable duration (sec), F0 s (Hz) and Jitter (%) through log-10 transformation method because these variables significantly deviated from normal distribution. The stepwise regression procedure was applied on 10 independent variables to exclude non-significant variables. After the extraction of important variables by the stepwise regression procedure, a cross validated (leave-one-out classification method) discriminant function analysis (DFA) was applied for the correct classification of the vocalization. In DFA analysis, the selected independent variables were used as a predictor, whereas type of call as a dependent grouping variable. This statistical analysis lets us to identify the most important variables for separation of the call types and to visualize the structural differences and similarities in the classification of call types [15]. The Wilk’s Lambda was used to measure the accuracy of correct classification of cases into different groups on the basis of discriminant functions. Three parameters viz., no. of full-length SEs, no. of half-length SEs and no. of diffused SEs were not included in the stepwise selection procedure as they could not be normalized with the data transformation methods. Henceforth, Kruskal-Wallis ANOVA was applied for differences among all the vocal categories followed by post-hoc Mann-Whitney U-tests (2—tailed) for multiple comparisons among groups. Holm’s sequential Bonferroni procedure with α = 0.05 was used for the correction of multiple-comparison Mann-Whitney U-test results [41]. The data are presented as mean ± S.E. P < 0.05 was considered statistically significant difference.

## Results

### Statistical classification

Descriptive statistics of 13 different vocal parameters for a total of 6 vocal categories are presented in Table 2. Eight out of ten parameters, chosen by stepwise regression model were Dur (s), F0 s (Hz), Shimmer (%), F0 max (Hz), F0 e (Hz), F0 min (Hz), Pitch max (Hz) and Pitch m (Hz). The first two functions in DFA showed maximum variance (Function 1: 63.9%, eigenvalue = 59.42; Function 2: 26.6%, eigenvalue = 24.75) and showed highly significant differences between the different types of calls (Wilks’ λ DF1/5 = 0.001, df = 40, P < 0.001 and Wilks’ λ DF2/5 = 0.001, df = 28, P < 0.001). The function one in standardized canonical discriminant function coefficients showed positive coefficients for duration (0.64), F0 e (0.58), and F0 max (0.41) and high negative coefficient for Pitch max (-0.90). Function two showed high positive coefficients for duration (0.76), and Pitch max (0.60; S1 Table).

**Table 2:** The descriptive statistics of acoustic parameters measured for all six vocal categories.

	Vocal category					
Acoustic parameter	Type 1 cooee (n = 108)	Type 2 cooee (n = 36)	Type 1 coegh (n = 40)	Type 2 coegh (n = 52)	Wurroo (n = 55)	Coe (n = 43)
Dur (s)	0.441±0.0006	0.468±0.009	0.397±0.005	0.411±0.004	0.162±0.0019	0.90±0.0032
F0 s (Hz)	13482±9.83	14356±29.05	15220±211.0	15220±20.05	14133±310.86	11487±25.93
F0 e (Hz)	13588±87.04	12055±94.42	22605±52.99	16995±35.29	13207±334.53	11488±34.06
F0 min (Hz)	13222±18.41	9004±44.40	13292±402.0	12997±31.59	12311±40.38	10765±18.54
F0 max (Hz)	19483±24.44	20539±60.76	23348±225.7	18441±35.22	19886±51.19	17824±28.20
Pitch m (Hz)	536±0.54	490±10.30	514±4.9	526±0.76	501±6.19	474±5.57
Pitch min (Hz)	442±0.74	299±1.84	362±1.89	451±1.26	409±1.20	381±1.06
Pitch max (Hz)	589±0.44	604±1.77	518±0.76	574±1.0	615±1.48	565±0.98
Jitter (%)	2.24±0.08	3.53±0.23	4.81±0.09	2.18±0.06	3.35±0.14	3.65±0.12
Shimmer (%)	6.4±0.25	9.17±0.40	14.72±0.55	9.87±0.29	13.47±0.55	13.67±0.41
No. of full-length SEs	9.19±0.18	4.36±0.28	0.0±0.0	0.0±0.0	5.84±0.36	5.61±0.10
No. of half-length SEs	0.86±0.07	4.67±0.34	0.0±0.0	10.21±0.18	2.24±0.14	2.30±0.14
No. of diffused SEs	0.0±0.0	0.94±0.22	10.2±0.14	5.11±0.09	0.0±0.0	0.0±0.0

Values are presented as mean ± S.E., n = number of calls.

**Fig 1:**
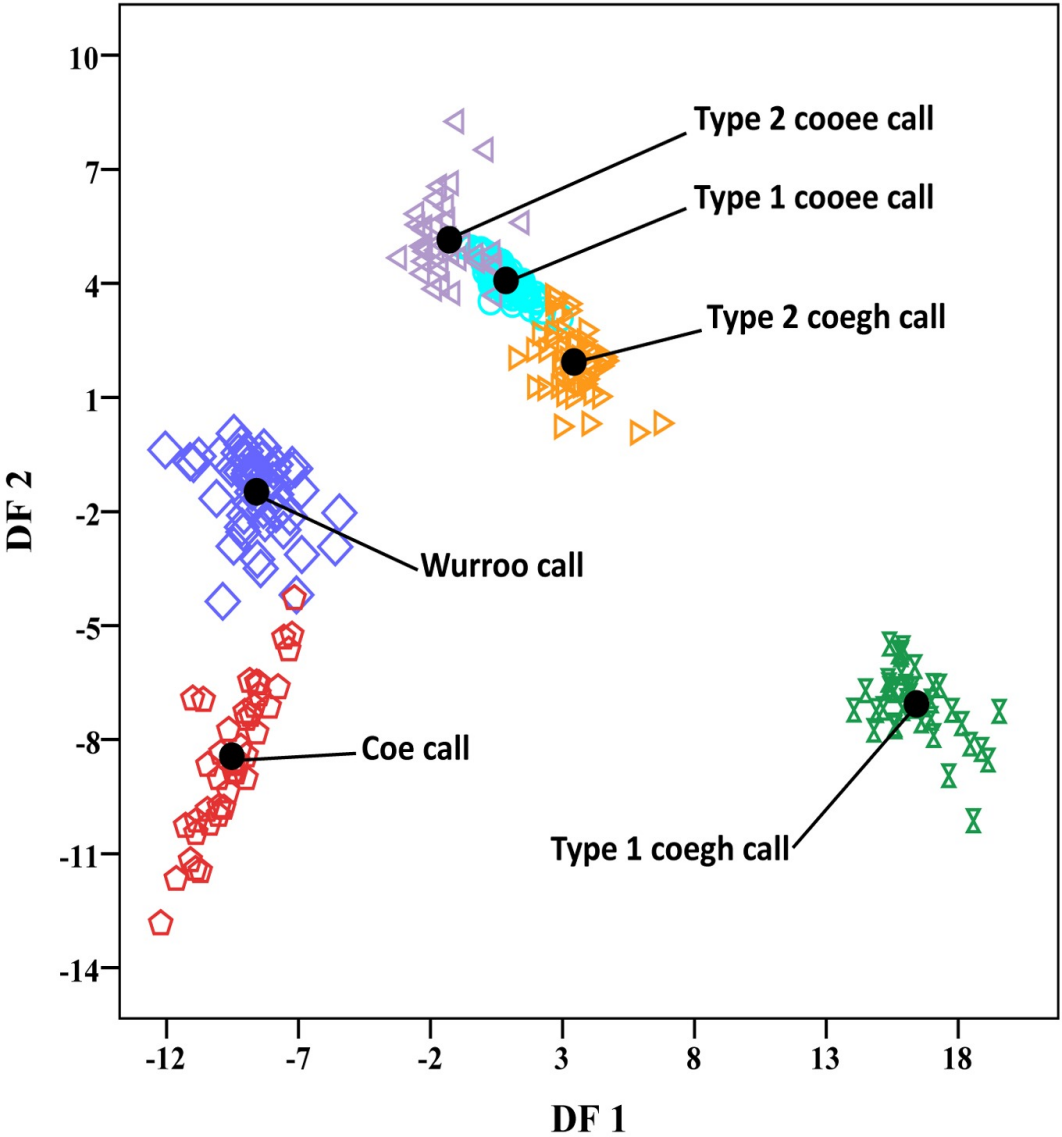
Plot of discriminant scores generated by discriminant function 1 and 2 which shows distinct separation of the six vocal types of the adult male Asian koel. Black dots show the centroids of the different vocal categories.

Using the cross validated method, DFA classified the vocalizations correctly to 100% into the predicted vocal categories that we initially classified on the basis of spectrographic examination. The DFA correctly classified 100% (108/108) of *type 1 cooee call*, 100% (36/36) of *type 2 cooee call*, 100% (40/40) of *type 1 coegh call*, 100% (52/52) of *type 2 coegh call*, 100% (55/55) of *wurroo call* and 100% (43/43) of *coe call* (S2 Table). All six vocal types were distinctively separated in space in the plot defined by discriminants function 1 and 2 scores (Fig 1). Moreover, the remaining three parameters (No. of full-length secondary elements, no. of half-length secondary elements and no. of diffused secondary elements) that were not used in DFA analysis, were also found significantly different among all the six vocal categories. The statistical description is presented in Table 3.

**Table 3:** Summary of statistical results of three nonnormalized parameters.

Vocal category comparison		Acoustic parameter								
		No. of full-length SEs H = 278.39, df = 5, P = 0.001			No. of half-length SEs H = 268.41, df = 5, P = 0.001			No. of diffused SEs H = 310.80, df = 5, P = 0.001		
		U	z	P	U	z	P	U	z	P
***Type 1 cooee* vs**	*Type 2 cooee*	85	-8.66	0.003	99	-8.89	0.003	1188	-6.78	0.003
	*Type 1 coegh*	0	-9.47	0.003	700	-7.0	0.003	0	-11.95	0.003
	*Type 2 coegh*	0	-10.45	0.003	0	-10.55	0.003	0	-12.31	0.003
	*Wurroo*	898.5	-7.35	0.004	973	-7.48	0.004	2970	0	0.05
	*Coe*	150	-9.08	0.004	586	-7.57	0.004	2322	0	0.025
***Type 2 cooee* vs**	*Type 1 coegh*	0	-8.17	0.005	0	-8.12	0.005	0	-7.65	0.004
	*Type 2 coegh*	0	-8.97	0.005	10	-7.92	0.005	0	-8.06	0.004
	*Wurroo*	704	-2.45	0.016	284	-5.91	0.006	605	-4.97	0.005
	*Coe*	482	-3.32	0.006	225	-5.51	0.007	473	-4.45	0.005
***Type 1 coegh* vs**	*Type 2 coegh*	1040	0	0.05	0	-8.60	0.008	0	-8.28	0.006
	*Wurroo*	20	-8.57	0.007	0	-8.76	0.01	0	-9.29	0.007
	*Coe*	0	-8.55	0.008	0	-8.38	0.012	0	-8.51	0.008
***Type 2 coegh* vs**	*Wurroo*	26	-9.40	0.01	0	-9.05	0.016	0	-9.63	0.01
	*Coe*	0	-9.32	0.012	0	-8.44	0.025	0	-8.83	0.012
***Wurroo* vs**	*Coe*	1171	-0.087	0.025	1149	-0.25	0.05	1182	0	0.016

Abbreviations: SEs = Secondary elements, H = Kruskal–Wallis ANOVA, df = degree of freedom, U = Mann- Whitney U -test, z = Wilcoxon W test and P = Probability.

### Classification and description of calls

**Fig 2:**
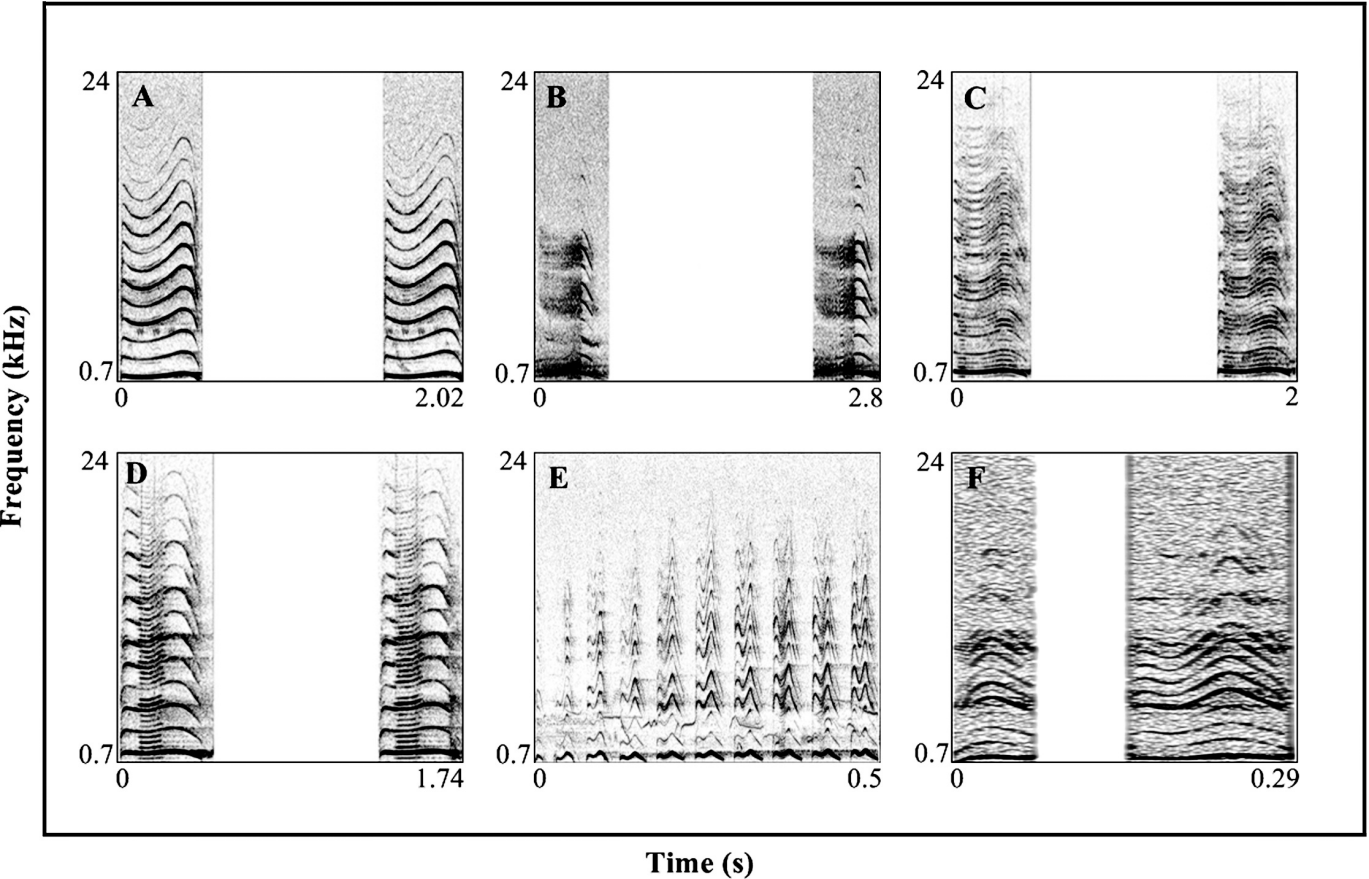
Spectrograms of different vocal categories of the adult male Asian koel. Two consecutive *type 1 cooee calls* (A), two consecutive *type 2 cooee calls* (B), two consecutive *type 1 coegh calls* (C), two connective *type 2 coegh calls* (D), a bout of *wurroo calls* (E), two consecutive *coe calls* (F). Spectrograms were constructed in Praat using the Fast Fourier Transform method (FFT), Gaussian window shape, window length of 0.012 s, frequency view range of 700–24000 Hz, pitch floor of 75–700 Hz, dynamic range of 70 dB, intensity range of 30–100 dB, 0.5 s zooming level.

A total of six distinctive vocal categories were identified on the basis of initial visual and audible inspection of the spectrograms and subsequent validation via Discriminant Function Analysis (Fig 2). For each of the vocal category, the description of the spectrographic pattern and various characteristics are described below:

#### Type 1 cooee call (S1 Audio)

The *type 1 cooee call* is a harmonically rich call compared with all other types of calls. It contained a primary element of high spectral energy which produced maximum number of full-length secondary elements that added maximum melodies to this call (Fig 2A). This call was found repeated in a continuous manner at dawn and dusk until a female bird joined the male bird’s territory. This call type was also the most common type (e.g. 205/750 or 33% calls emitted by koels were of this call type) of the adult male Asian koel vocalizations and pleasant to human hearing. The shimmer (%) value for this call was found to be lower than all other type of calls (Table 2). The *type 1 cooee call* also overlapped slightly with *type 2 cooee call* and *type 2 coegh call* in DFA analysis (Fig 1), statistical analysis separated them (Table 3) whereby, the former was most common and the latter two were less common, thus further distinguishing these three call types.

#### Type 2 cooee call (S2 Audio)

This call type had fewer full-length secondary elements compared to the *type 1 cooee call*, while some of the secondary elements showed a diffused pattern (Fig 2B). The birds emitted these calls continuously at midday or at the end of dawn without including any other type of call in a bout or sometimes at the start of a bout which contained the *type 1 cooee calls*, *type 1 coegh calls* and *type 2 coegh calls*. The jitter and shimmer percent of this call had higher values compared to the *type 1 cooee call* (Table 2). This call type was frequently produced by the bird when it had produced *type 1 cooee calls* for a long time or in a situation where other birds like crows and male birds of same species entered its territory.

#### Type 1 coegh call (S3 Audio)

All the secondary elements which were produced by the primary element in this call type were diffused which clearly visually distinguished them from other type of calls. This call type had no distinguishing half-length or full-length secondary elements (Fig 2C). Jitter and shimmer percent values were higher in comparison to all other calls (Table 2). This type of vocalization was sharp and unpleasant to human hearing. The bird uttered this call in the middle of a bout of *type 1cooee calls* when it usually produced the latter calls continuously for up to 8.0 ± 2.0 minutes.

#### Type 2 coegh call (S4 Audio)

Spectrographic examination of *type 2 coegh calls* confirmed that the first and last parts of the primary element were not involved in the production of clear-cut secondary elements. In contrast the middle half portion of the primary element in the syllable produced clear secondary elements of half-length (Fig 2D). The jitter value was lower as compared to all other call types (Table 2). This vocalization was emitted by the birds in a bout usually before the *type 1 coegh call* or in between a long bout of the *type 1 cooee calls*. Similar to *type 1 coegh call*, the birds never started their bouts from *type 2 coegh calls*. Spectrographically both type 1 and type 2 *coegh calls* could be visibly distinguished from each other.

#### Wurroo call (S5 Audio)

Koels frequently produced *wurroo calls* in a continuous manner. Except the *coe call*, these calls were of shorter duration and were produced less frequently as compared to all other types of calls. The starting two calls in the bout of *wurroo calls* were usually short and produced fewer full-length secondary elements, while the latter calls had long length durations and produced maximum secondary elements of full-length. The full-length secondary elements in syllables of the starting *wurroo calls* had low spectral energy as compared to the full-length secondary elements of the middle *wurroo call* in a *wurroo call*s bout (Fig 2E). This vocalization was produced usually at early dawn and sunset and most often the birds used to take start of their vocalizations with this call type.

#### Coe call (S6 Audio)

This type of vocalization was of shorter duration and was found to be produced at times when other male or female birds of same species perched on the same tree. The call type was more frequently heard when the birds were involved in quick communications and foraging. When calling, the birds were found usually moving to and fro on the branches of a tree. In comparison to other type of calls, the F0s (Hz) and F0e (Hz) values of *coe call* were very low (Fig 2F, Table 2).

## Discussion

The present study is the first to provide a comprehensive acoustic analysis of adult male Asian koels vocalizations. We recorded and analyzed a total of 334 high quality calls recorded in its wild habitat in two consecutive breeding seasons and determined six distinct call types in the vocalizations.

Previously Maller and Jones [29] identified three different vocal categories for the male adult common koel; “*cooee*”, “*wurroo*” and “*whik*”. In our findings the spectrograms of *type 1 cooee call*; the most common call type, appeared substantially different from the *cooee call*, the most common call type in their findings. For example, greater numbers of secondary elements are produced by the primary high spectral element in this type of call. We were so far unable to record any *whik call* from the adult Asian koel in two consecutive breeding seasons. Instead, we identified four other call types that we categorized as; “*type 2 cooee*”, “*type 1 coegh*”, “*type 2 coegh*” and “*coe*”, and also measured various structural and temporal parameters for all call types. Differences in the vocalization of koels found in Asia and Australia support the possibility of either species or sub-species differences among these geographically distinct populations. Although, the taxonomy of common koel is complex, both the findings of Maller and Jones [29] and ours provide useful evidences for systematic clarification of the common koel in future studies. Though, it has also been reported that common koels found in Australasia have similar calls [26]. The present study thus further reveals that the Asian koel produces six different vocalizations, while the koels found in Australia produce only three kinds of vocalizations [29]. In addition, like the most common call type “*cu-coo*” of the common cuckoo, previously studied for vocal distinctiveness in detail [42–44], the Asian koel also frequently produced the “*type 1 cooee call”* in its vocalizations. Future studies to examine this call type for intra and inter individual variations would be valuable.

The DFA results from 10 call parameters accurately classified 100% of the adult male Asian koel calls according to the predicted vocal categories which were identified on the basis of visual examination of spectrograms. These results were relatively higher than the previously reported vocal classification for birds: e.g. 90.5% in case of African penguins [31], 83.3% in case of great curassows (*Crax rubra*) [45], and 74.2% in the case of the smooth-billed anis (*Crotophaga ani*) [15]. Furthermore, in the statistical analysis of the three acoustic parameters (No. of full-length SEs, no. of half-length SEs and no. of diffused SEs) both the Mann-Whitney U-test (two—tailed) and Wilcoxon W test results were taken into consideration because the latter one is also considered a different version of Mann-Whitney U-test in SPSS software analysis [46].

The current study also confirms that, all the types of calls (*type 1 cooee*, *type 2 cooee*, *type 1 coegh*, *type 2 coegh*, *wurroo and coe call*) of the adult male Asian koel are harmonically rich calls, their syllables consist of a basic primary element with nearly similar fundamental frequency range but the secondary elements differ in composition and number. It has been described previously, that sounds which have frequencies occuring together add melody to the sound and are basically the multiples of the first element frequency and can be produced by a single source [11, 47]. Besides, some syllables contain structures which are not harmonically related to each other and may originate from separate sources of the sound [48]. Our study provides evidence that in the vocalization of the adult male Asian koel, the primary elements are harmonically connected with the secondary elements in the syllables of all types of calls. This is so because during spectrogram analysis we found a reduction in the number of secondary elements that coincided with a frequent decrease in the spectral energy of the primary element.

Most of the host bird species employ various tactics, such as high level of rejection toward cuckoo eggs or high level of aggressiveness toward the female parasite to escape brood parasitism [49]. The aggression of the host toward the brood parasites has already been reported in common cuckoo [50] and several African cuckoos [51]. Parasitic cuckoos however use the strategy to evolve quickly to overcome resistance by their host species [49]. Furthermore, birds also show aggression by producing usually low pitched [52, 53] and harsh vocalizations [54, 55]. It is clear from our findings that *type 1 coegh call* and *type 2 coegh call* lack harmonics, have low pitch values and, contain more noise compared to the *type 1 cooee call*. These findings indicate that the adult male Asian koel may use these call types to display some kind of aggression to compete efficiently with its host species to well utilize its short breeding season in order to achieving an ultimate high breeding success. It also came to our observation that during the courtship display, male koels got agitated, became more vocal on seeing a female koel and began spectacular chases toward the females. In addition, the *type 1 cooee call* was frequently produced by the birds during the whole day and occasionally also at night time. Again, this type of activity indicates that this call type is most likely the advertising call. However, assigning functions to calls was beyond the scope of the present study and therefore need extensive additional work.

In conclusion our study provides first comprehensive description of various acoustic parameters and the statistical validation of the adult male Asian koels vocalization recorded in their breeding seasons. Although current findings are limited only to the breeding season and did not fully explore the entire vocalizations of the male Asian koel, still we feel confident that we recorded all type of vocalizations of the adult male Asian koel in their breeding season. This is so because we recorded the birds i) from initial arrival in Pakistan till the start of back journey toward India, ii) three times in a day, that is at early dawn, noon and at dusk. However, we consistently found that noon time recordings had greater noise. To our understanding this was due to the reason that several other bird species were active at these times. We did initial analyses on these recordings to identify any other potential vocal category, but we found that most of the vocal categories were similar to those identified at dawn and dusk time recordings. Thus these recordings were omitted from further analyses.

Our findings therefore enrich the biological knowledge about the adult male the Asian koel vocalizations and provide a foundation for future comparative studies among different species of other cuckoos. Future studies should focus on exploring the entire vocal repertoire of male Asian koels and to understand the functions of the vocalizations in sexual selection, social interaction and individual recognition.

## Supporting information

S1 Audio Three *type 1 cooee calls* uttered by three different birds. (WAV)

S2 Audio Three *type 2 cooee calls* uttered by a single bird. (WAV)

S3 Audio Two *type 1 coegh calls* uttered by a single bird. (WAV)

S4 Audio Two *type 2 coegh calls* uttered by two different birds. (WAV)

S5 Audio A bout of *wurroo calls* uttered by a single bird. (WAV)

S6 Audio Four *coe calls* uttered by two different birds. (WAV)

S1 File Raw data. (XLSX)

S1 Table Standardized canonical discriminant function coefficients values of total eight parameter extracted by stepwise regression model procedure used in discriminant function analysis. (DOC)

S2 Table Classification results of the stepwise cross validated discriminant function analysis. (DOC)
