# Supplementary material for: Vocalizations of adult male Asian koels (Eudynamys scolopacea) in the breeding season
Source: PLoS One. 2017 Oct 20;12(10):e0186604. doi: 10.1371/journal.pone.0186604 (PMC5650150; doi:10.1371/journal.pone.0186604)
Supplement: S1 Table — (DOC) [file pone.0186604.s008.doc]

Table S1. Standardized canonical discriminant function coefficients values of total eight parameter extracted by stepwise regression model procedure used in discriminant function analysis.

Standardized Canonical Discriminant Function Coefficients	
	Function	
	1	2	3	4	5	
d_log10	.644	.758	-.042	-.116	.050	
fs_log10	.195	-.043	.242	.220	.554	
sh	.032	-.105	.356	.163	.336	
fmx	.410	-.314	.769	-.123	-.496	
fe	.578	-.269	-.037	.459	.408	
fmn	.126	.067	-.288	.655	-.614	
pmx	-.895	.602	.394	.386	.075	
pm	.223	-.115	-.335	-.141	-.185	
